# Effect of VN and TiB_2_-TiC_x_ Reinforcement on Wear Behavior of Al 7075-Based Composites

**DOI:** 10.3390/ma14123389

**Published:** 2021-06-18

**Authors:** Yaping Bai, Jiale Wei, Naqing Lei, Jianping Li, Yongchun Guo, Mengmeng Liu

**Affiliations:** School of Materials and Chemical Engineering, Xi’an Technological University, Xi’an 710021, China; 18821777031@163.com (J.W.); leinaqing@163.com (N.L.); nianbai1116@163.com (J.L.); 18706752776@163.com (Y.G.); qwelmm_123@163.com (M.L.)

**Keywords:** VN/7075 composites, TiB_2_-TiC_x_/7075 composites, microstructure, hardness, friction and wear properties

## Abstract

Al 7075 alloy, 15 wt.% VN/7075 composites, and 20 wt.% TiB_2_-TiC_x_/7075 composites were prepared by ball milling with subsequent hot-pressing sintering. The microstructure, hardness, and wear properties at room temperature to 200 °C of Al 7075-based composites with different reinforcement phases were discussed. The grain uniformity degree values of 15 wt.% VN/7075 composites and 20 wt.% TiB_2_-TiC_x_/7075 composites were 0.25 and 0.13, respectively. The reinforcement phase was uniformly distributed in 15 wt.% VN/7075 composites and 20 wt.% TiB_2_-TiC_x_/7075 composites, almost no agglomeration occurred. The order of hardness was 20 wt.% TiB_2_-TiC_x_/7075 composites (270.2 HV) > 15 wt.% VN/7075 composites (119.5 HV) > Al 7075 (81.8 HV). At the same temperature, the friction coefficient of 15 wt.% VN/7075 composites was the lowest, while the volume wear rate of 20 wt.% TiB_2_-TiC_x_/7075 composites was the lowest. With the increase of temperature, the wear mechanism of Al 7075 changed from spalling wear to oxidation wear and adhesion wear. However, the wear mechanisms of 15 wt.% VN/7075 and 20 wt.% TiB_2_-TiC_x_/7075 composites changed from abrasive wear at room temperature to wear mechanism (oxidation wear, abrasive wear, and adhesive wear) at medium and low temperature. Comprehensive wear test results indicated that 20 wt.% TiB_2_-TiC_x_/7075 composites had excellent tribological properties at medium and low temperature.

## 1. Introduction

With the rapid development of science and technology, the demand for light weight, high strength, and excellent functional materials in the field of military and civil equipment is gradually increasing, which promotes the rapid development of metal matrix composites [1,2,3]. Among them, aluminum matrix composites (AMCs) have shown a broad application prospect in aerospace, automobile, and power electronics due to their high specific strength, specific modulus, low density, low coefficient of thermal expansion, good dimensional stability, excellent thermal and conductive properties, as well as their structure and functionality [4,5,6]. As for the engine sliding components made of aluminum matrix composites, such as pistons, it is one of the core components of the engine. The main operating conditions are medium and low temperature environment (generally, the operating temperature of the piston is less than 300 °C), which is subjected to huge mechanical loads and thermal loads [7,8]. During the operation process, the increase of wear contact surface temperature leads to the formation of some oxidation products with lubricating properties [9,10,11]. The hardness of substrate material is too low to support the surface oxide film, while it is difficult to machine the parts because of the hard base material. Therefore, research on the reasonable matching between the mechanical properties of the matrix and its surface oxide lubrication properties has a certain practical value.

7075 aluminum alloy with Al, Zn, Mg, and Cu elements as the main element has low density, excellent strength, good processability, high toughness and corrosion resistance, which has the potential to be used as engine transmission parts. However, its wear resistance is relatively poor under actual working conditions [12,13]. For metal matrix composites, the addition of reinforced phase can bear the main load, and it can also produce fine grain strengthening to the matrix, thereby improving the properties of the matrix metal [14,15]. At present, experts and scholars at home and abroad have shown that the mechanical properties of materials can be greatly improved by adding cemented carbide particles and ceramic particles. Therefore, adding a particle-reinforced phase is an effective way to improve the wear resistance of 7075 aluminum alloy [16,17,18].

VN ceramic particles not only have high hardness and good ductility, but also have good physical and chemical compatibility with 7075 aluminum alloy [3,19]. The addition of VN can significantly improve the hardness of the material, and VN can be oxidized to form V_2_O_5_ and VO_2_ lubricating phases at high temperature, which can improve the wear properties of the material [20,21]. Two reinforcing phases of TiB_2_ and TiC have excellent hardness, wear resistance, corrosion resistance, and thermal stability, in addition to good wettability with aluminum matrix, while also not reacting with aluminum [22,23]. Mandal et al. [24] prepared in-situ TiB_2_/Al-4Cu composites, and the wear test results under dry friction condition showed that the formation of TiB_2_ phase with high hardness and good adhesion improves the friction and wear properties of Al-4Cu alloy. By comparing the friction and wear properties of TiC_x_/Al composites and hypereutectic Al-Si alloy under dry friction condition, Shu et al. [25] found that the wear resistance of TiC_x_/Al composites was much higher than that of Al-Si alloy. Han et al. [26] found that adding TiB_2_-TiC_x_ particles into the metal matrix can reduce the casting defects and improve the metallurgical quality, thus significantly improving the mechanical properties and wear properties of the material. However, the comprehensive properties of composites are closely related to their microstructure uniformity, therefore, it is very important to study the microstructure uniformity before characterizing the properties of composites. Edward Bormashenko et al. [27] reviewed the characterization of self-assembled 2D patterns with Voronoi entropy, and concluded that the Voronoi entropy was a mathematical tool for quantitative characterization of the orderliness of points distributed on a surface. Wang [28] put forward a new concept of grain size uniformity and established the corresponding formula based on the comprehensive analysis of the grain structure image of steel.

In conclusion, VN and TiB_2_-TiC_x_ particles can be introduced into 7075 aluminum alloy matrix to develop aluminum matrix composites with excellent microstructure, mechanical properties, and high wear resistance. According to the previous research on the wear properties of different contents of VN reinforced Al 7075 matrix composites, it is found that 15 wt.% VN/7075 composites had excellent friction and wear properties in sulfur atmosphere [29]. Besides, TiB_2_-TiC_x_/7075 composites have good comprehensive room temperature mechanical performance when TiB_2_-TiC_x_ content was 20 wt.% [30]. Therefore, 15 wt.% VN/7075 composites and 20 wt.% TiB_2_-TiC_x_/7075 composites were prepared to study the wear mechanism of the composites at different temperatures (room temperature, 100, and 200 °C), which provides a theoretical basis for the further application.

## 2. Experimental Procedures

Al 7075 powders, Ti powders, B_4_C powders (produced by Changsha Tianjiu company, Changsha, China) and VN particles (produced by Naiyate alloy Welding Materials Co., Ltd., Nangong, China) were used as raw materials to fabricate the VN/7075 aluminum matrix composites and TiB_2_-TiC_x_/7075 aluminum matrix composites. A total of 36 g 7075 powders and 6 g VN particles were blended in a planetary ball-mill (YXQM-4L, Changsha MITR Instrument & Equipment Co., Ltd., Changsha, China) at the speed of 100 r/min for 5 h. The ball-to-power weight ratio was selected to be 3:1, the grinding atmosphere was argon (Xi’an TEDA cryogenic equipment Co., Ltd., Xi’an, China), and the process control agent was stearic acid (5% of total mass, Tianjin Tianli Chemical Reagent Co., Ltd., Tianjin, China). After the milling process, the VN/7075 composites powders were loaded into a graphite mold (Xianyang Kejia Machinery Equipment Co., Ltd., Xianyang, China) with a diameter of 30 mm and purity of 99% for sintering by ZT-40-20Y vacuum hot-pressing furnace (Shanghai Chenhua Technology Co., Ltd., Shanghai, China). The vacuum degree was 10^−1^–10^−3^ Pa and the pressure was 20 MPa. The sintering temperature was 520 °C for 120 min, and then cooled to room temperature with furnace cooling. The sintered sample size was about: φ 30 mm × 10 mm.

The TiB_2_-TiC_x_ composites powders preparation process was as follows: 27.46 g Ti powders and 10.54 g B_4_C particles were milled in a planetary ball-mill and 38 g TiB_2_-TiC_x_ composite powders (theoretical value) was produced through self-propagating high-temperature synthesis reaction induced by mechanical alloying. The ball milling parameters of 3Ti + B_4_C→2TiB_2_ + TiC are shown in Table 1. Then, 8 g TiB_2_-TiC_x_ composites powders and 32 g Al 7075 powders were used as the raw material of TiB_2_-TiC_x_/7075, and the mixing process and hot-pressing sintering process were same as the preparation of VN/7075 composites.

The phase structure was analyzed by XRD-6000 X-ray diffractometer (XRD, Shimadzu, Tokyo, Japan, in which the scanning speed was 4°/min, the range was 20°–90°, the speed length was 0.02) using Cu Kα radiation. Microstructure and element distribution of three materials was studied using VEGA II XMU Scanning Electron Microscope (SEM, TESCAN, Brno, Czech Republic). The micro-hardness of the composite samples was measured by the Vickers hardness (402MVD, Instron, Norwood, USA) method under a load of 200 g for a dwell time of 15 s.

Wear behavior of three materials at room temperature, 100, and 200 °C were examined by high temperature friction and wear tester (HT-1000, Lanzhou Zhongke Kaihua Technology Development Co., Ltd., Lanzhou, China). The friction contact mode was ball-on-disk. The disk specimens, with a size of φ 20 mm × 2 mm, were made of Al 7075, 15 wt.% VN/7075, and 20 wt.% TiB_2_-TiC_x_/7075 composites; the ball specimens, with a size of 4 mm in diameter, were made of Al_2_O_3_. Wear properties were tested under a load of 30 N for 30 min, and the frequency was 3 Hz. Before the experiment, wear samples should be polished with sandpaper.

The friction coefficients of materials were obtained using the friction coefficient measurement module of the wear tester. The volume of wear scar contour was measured by 3D laser confocal scanning microscope (VK-9710K, Keyence, Osaka, Japan), and the wear rate was calculated by Equation (1). Each group of tests was repeated three times, and the average value of friction coefficient and wear rate was taken.

(1) $$w=\frac{\Delta V}{F\cdot L}$$

where *w* is the wear rate (10^−3^·mm^3^·N^−1^·m^−1^), Δ*V* is the volume loss (μm^3^), *F* is the applied load (N), and *L* is the sliding distance (mm).

The worn surface morphology was observed by VEGA II XMU scanning electron microscope (SEM, TESCAN, Brno, Czech Republic), the composition of worn surface was analyzed by Energy Dispersive Spectroscopy (EDS, Oxford Instruments, Oxford, UK). The composition of the worn surface at different temperatures was analyzed by Invia confocal laser Raman spectrometer (Renishaw, Gloucestershire, UK). The wavelength was 785 nm and the collection range was 200–2000 cm^−1^. The morphology, element distribution of the worn surface, and cross-section layer were analyzed by SEM, and the wear mechanism of the three materials at different temperatures was analyzed.

The particle reinforcement phase of Al 7075, VN/7075 composites and TiB_2_-TiC_x_/7075 composites was marked with yellow color and its area was measured. The area of single particle is $A_{i}$. Combining the particle area with the equal area circle area method, the target particle size $d_{i}$ was calculated. The calculation formula is as follows.

(2) $$d_{i}=2\sqrt{\frac{A_{i}}{\pi }}$$

The microstructure distribution of composites was characterized by the characteristic parameter of grain uniformity degree (*GME*) [28]. It was defined as the ratio of the standard deviation to the expected value of all grain sizes in the image. The calculation formula is shown in Equation (3), where $\sigma_{d}\;$ is the standard deviation of grain size, $\mu_{d}\;$ is the expected value of grain size. The smaller the value of the *GME* is, the better the uniformity of the grain distributed.

(3) $$GME=\left|1-\frac{\sigma_{d}}{\mu_{d}}\right|$$

(4) $$\sigma_{d}=\sqrt{\frac{\sum_{i=1}^{n}{\left(d_{i}-\mu_{d}\right)}^{2}}{n-1}}$$

(5) $$\mu_{d}=\frac{1}{n}\overset{n}{\underset{i=1}{\sum }}d_{i}$$

## 3. Results and Discussions

### 3.1. Microstructure Analysis

**Figure 1:**
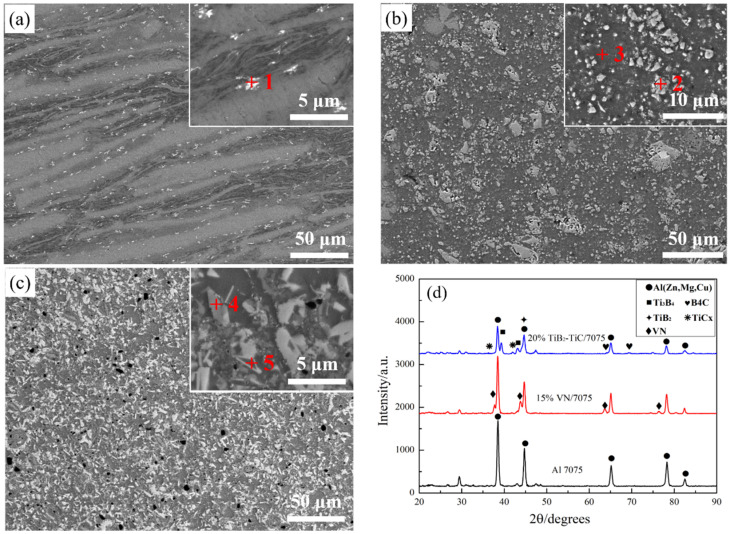
Microstructure and phase analysis of samples. (**a**) Al 7075; (**b**) 15 wt.% VN/7075; (**c**) 20 wt.% TiB_2_-TiC_x_/7075; (**d**) XRD pattern.

**Figure 2:**
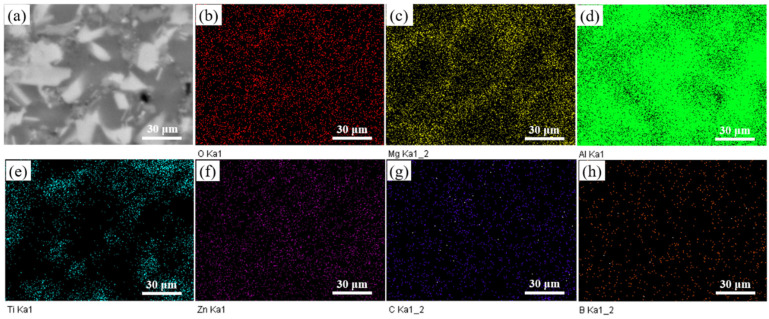
Alloying elements distribution of 20 wt.% TiB_2_-TiC_x_/7075 composites. (**a**) micromorphology; (**b**) O; (**c**) Mg; (**d**) Al; (**e**) Ti; (**f**) Zn; (**g**) C; (**h**) B.

**Figure 3:**
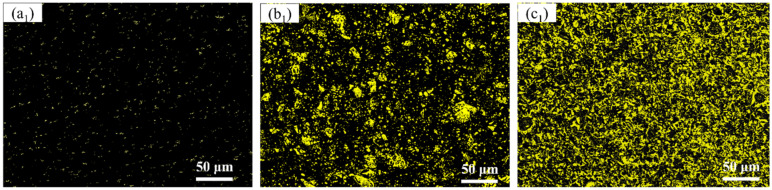
Image after region calibration. (**a1**) Al 7075; (**b1**) 15 wt.% VN/7075; (**c1**) 20 wt.% TiB_2_-TiC_x_/7075.

Microstructure and XRD patterns of Al 7075, 15 wt.% VN/7075, and 20 wt.% TiB_2_-TiC_x_/7075 composites are present in Figure 1, respectively. Table 2 presents EDS results (wt.%) in regions 1–5 indicated in Figure 1a–c. Figure 2 is the alloying elements distribution of 20 wt.% TiB_2_-TiC_x_/7075 composites. The calibration microstructures of Al 7075, 15 wt.% VN/7075 composites, and 20 wt.% TiB_2_-TiC_x_/7075 composites are shown in Figure 3. It can be seen from Figure 1a that Al 7075 alloy is mainly composed of gray matrix phase and white dots. Combining XRD results in Figure 1d and phase analysis in Table 2, it can be determined that gray matrix phase and white dots are Al (Zn, Mg, Cu) and copper rich phase, respectively. Combined with Figure 1b and Table 2, it can be concluded that the white particles in region 2 are VN in 15 wt.% VN/7075 composites, and the VN particles are evenly distributed without any agglomeration phenomenon. O element appears in region 3, which is caused by the low oxygen partial pressure during sample preparation (ball milling and hot-pressing sintering). Combined with Figure 1c and Figure 2 and Table 2, it can be concluded that region 4 is the mixture of Ti_3_B_4_ and TiC_x_, and region 5 is TiC_x_. The *GME* of three composites in Figure 3 were calculate by Equations (2)–(5) and the values are 0.33 (Al 7075), 0.25 (15 wt.% VN/7075 composites), and 0.13 (20 wt.% TiB_2_-TiC_x_/7075 composites), respectively. It can be deduced that the microstructure distribution of the three materials is relatively uniform because the *GME* values are small [28].

### 3.2. Hardness

The Vickers hardness results show that: the order of hardness is 20 wt.% TiB_2_-TiC_x_/7075 (270.2 HV) > 15 wt.% VN/7075 (119.5 HV) > Al 7075 (81.8 HV). Comparing with Al 7075, the hardness of 20 wt.% TiB_2_-TiC_x_/7075 and 15 wt.% VN/7075 composites are increased by 230.3 and 46.1%, respectively. This is due to the addition of the hard phase (TiB_2_-TiC_x_ or VN) and its uniform distribution.

### 3.3. Analysis of Wear Behavior

**Figure 4:**
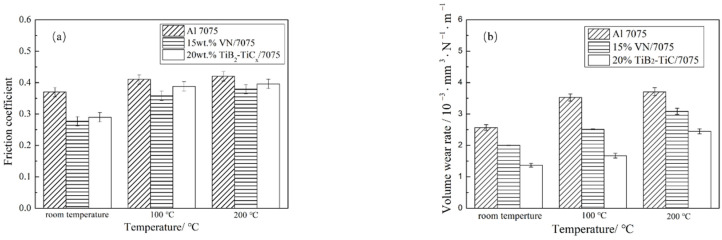
Friction coefficient and wear rate of samples after wear test at different temperatures. (**a**) friction coefficient; (**b**) wear rate.

The variation of friction coefficient and volume wear rate of Al 7075, 15 wt.% VN/7075 and 20 wt.% TiB_2_-TiC_x_/7075 composites with temperature are shown in Figure 4. Both the friction coefficient and volume wear rate of the three materials increase with the temperature increasing. At the same temperature, the friction coefficient and the volume wear rate of 15 wt.% VN/7075 and 20 wt.% TiB_2_-TiC_x_/7075 composites are lower than those of Al 7075. It also indicates that the friction coefficient of 15 wt.% VN/7075 composites is the smallest at all temperatures in Figure 4a. Compared with Al 7075, it reduced by 25.1 (room temperature), 12.7 (100 °C), and 9.8% (200 °C), respectively. This is because the crystal structure of VN is a face-centered cubic crystal and the V-N bond energy is weak, which means the V-N bond can be destroyed under small shear force [31]. Figure 4b reveals that the volume wear rate of 20 wt.% TiB_2_-TiC_x_/7075 composites is the smallest at all temperatures. Compared with Al 7075, it reduced by 46.2 (room temperature), 52.6 (100 °C), and 34.0% (200 °C), respectively. This is attributed to TiB_2_-TiC_x_ having high hardness and uniformly distributed in the matrix material, which improves the hardness of the matrix material and supports the matrix material better to resist the cutting of the abrasive material [32].

**Figure 5:**
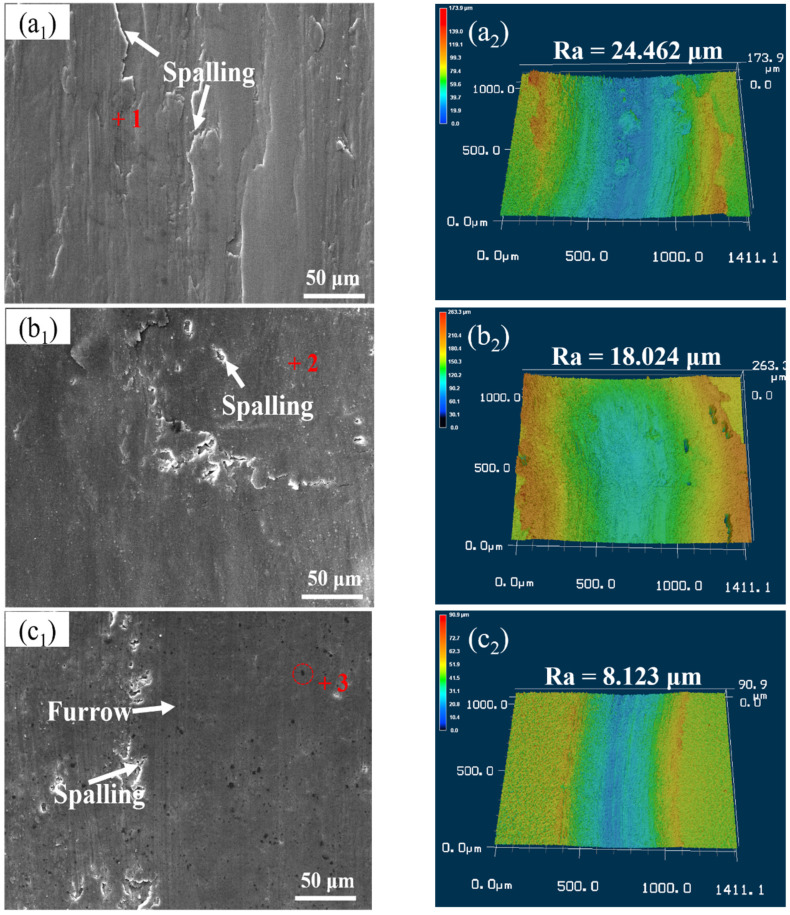
Worn surface analysis of samples after wear test at room temperature. (**a1**) Worn surface of Al 7075; (**a2**) 3D laser scheme 7075. (**b1**) Worn surface of 15 wt.% VN/7075; (**b2**) 3D laser scanning topography of 15 wt.% VN/7075; (**c1**) worn surface of 20 wt.% TiB2-TiCx/7075; (**c2**) 3D laser scanning topography of 20 wt.% TiB2-TiC_x_/7075.

The worn surface and 3D laser scanning morphology analysis of samples after wear test of Al 7075, 15 wt.% VN/7075, and 20 wt.% TiB_2_-TiC_x_/7075 composites at room temperature are shown in Figure 5. Table 3 shows the EDS results (wt.%) in regions 1–3 indicated in Figure 5. From Figure 5(a1), it can be seen that there is obvious spalling on the worn surface of Al 7075 (region 1). The worn surface of 15 wt.% VN/7075 composites has shallow micro-furrows in Figure 5(b1), which is formed by a small amount of wear particles pressed. White VN particles are uniformly distributed (region 2), which improves the hardness of the matrix and plays a good role in reducing wear. The worn surface of 20 wt.% TiB_2_-TiC_x_/7075 composites is smooth in Figure 5(c1) and there are shallow furrows and a few spalling pits. The hard black convex particles on the worn surface are TiB_2_ (region 3), which can protect the matrix during wear. Combined with Figure 5 and Table 3, it can be seen that there is micro-oxidation on the worn surface of the three materials, and the oxidation product is oxide of aluminum. This reveals that the wear mechanism of Al 7075 at room temperature is spalling wear, and 15 wt.% VN/7075 and 20 wt.% TiB_2_-TiC_x_/7075 composites are abrasive wear at room temperature.

The hardness of Al 7075 alloy is lower than Al_2_O_3_, so the plastic deformation is easier to occur on the surface of Al 7075 during the relative friction sliding process. This will cause the cracks and holes, which will further expand under the external force, making the material on the worn surface fall off and form flake debris. The reinforcement distributed uniformly improves the mechanical properties of the material and plays a supporting role in the wear process. This can resist the cutting of abrasive material effectively and protect the matrix material from wear. It is precisely because of the “supporting effect” and “shielding effect” that the wear resistance of the two composites is improved [33]. From Figure 5(a2–c2), it can be seen that the worn surface roughness (Ra) of 15 wt.% VN/7075 and 20 wt.% TiB_2_-TiC_x_/7075 composites at room temperature is 26.3 and 66.8% lower than that of Al 7075, respectively. This is consistent with the wear rate results in Figure 4.

**Figure 6:**
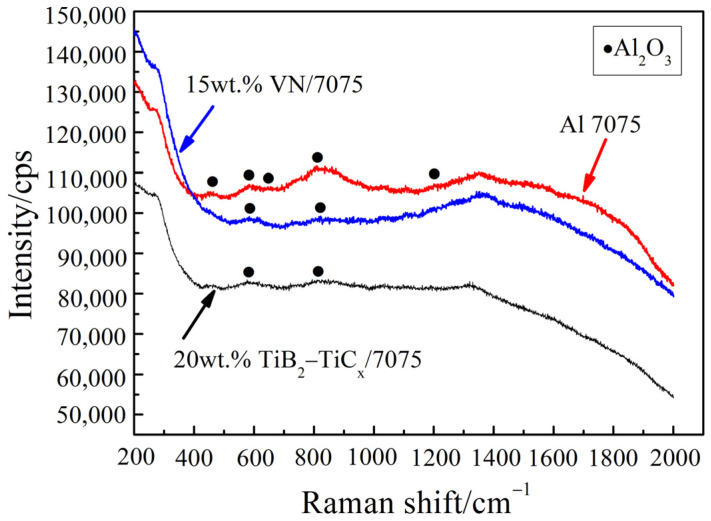
Raman spectrum test results of sample worn surface after wear test at room temperature.

In order to further judge whether there is oxidation wear at room temperature, the worn surface was tested by Raman spectroscopy, as shown in Figure 6. It can be seen from Figure 6 that Al_2_O_3_ appear on the room temperature worn surfaces of the three materials, which are due to the micro-oxidation caused by high flash temperature during friction and wear process. This is because the real contact of metal pair is only some higher convex bodies on the surface during dry sliding wear, so the stress on the real contact point of friction surface is much higher than the nominal contact stress. It results in the actual temperature higher than the average temperature of the overall friction surface, and the temperature of the local contact point becomes the flash point temperature (its value is much higher than the ambient temperature) [34]. The results of Raman spectroscopy are consistent with those of EDS shown in Table 3.

**Figure 7:**
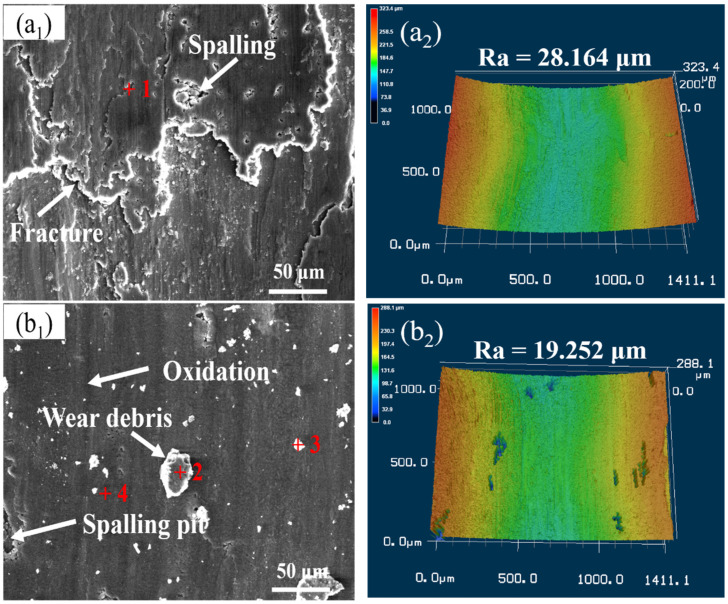

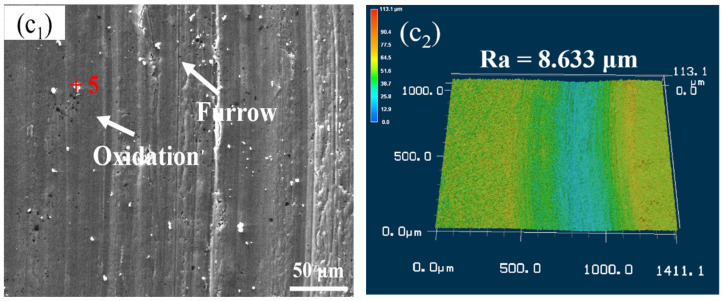
Worn surface analysis of samples after wear test at 100 °C. (**a1**) worn surface of Al 7075; (**a2**) 3D laser scanning topography of Al 7075; (**b1**) worn surface of 15 wt.% VN/7075; (**b2**) 3D laser scheme 15. wt.% VN/7075; (**c1**) worn surface of 20 wt.% TiB2-TiCx/7075; (**c2**) 3D laser scanning topography of 20 wt.% TiB_2_-TiC_x_/7075.

The worn surface and 3D laser scanning morphology analysis of samples after wear test of Al 7075, 15 wt.% VN/7075, and 20 wt.% TiB_2_-TiC_x_/7075 composites at 100 °C are shown in Figure 7, and Table 4 lists the EDS results (wt.%) in regions 1–5 indicated on Figure 7. It can be seen from Figure 7(a1–c1) and Table 4 that the worn surface of Al 7075 has a large and flat overburden layer (region 1), cracks and spalling pits with obvious plastic deformation characteristics, and that aluminum oxide is formed on the worn surface.

The worn surface of 15 wt.% VN/7075 composites is smooth and there are a small number of peeling pits, some black enamel layers (region 4) and block debris (regions 2 and 3). The oxides in the debris are mainly aluminum oxides and a small amount of vanadium oxides. There are a lot of furrows (region 5) and some abrasive grains on the worn surface of 20 wt.% TiB_2_-TiC_x_/7075 composites, and there are aluminum oxides in the worn surface. This indicates that the main wear mechanisms of Al 7075 at 100 °C are adhesive wear and oxidation wear, and 15 wt.% VN/7075 and 20 wt.% TiB_2_-TiC_x_/7075 composites are abrasive wear and oxidation wear at 100 °C.

At 100 °C, the radiation heat dissipation is not easy to carry out because of the high ambient temperature. Part of the friction heat of Al 7075 is taken away by peeling off, and the others will be transferred from the worn surface to the interior of the material, which makes the material internal temperature increased. This leads to a slight decrease in hardness of Al 7075. The mechanical properties of the friction pair Al_2_O_3_ are stable. Al 7075 and friction pair Al_2_O_3_ slide relatively, which causes plastic deformation and the microcracks will occur. The microcracks will expand, and lead to the peeling off of the worn surface under the external force. Friction heat will increase the instantaneous temperature of the worn surface, resulting in varying degrees of oxidation of the worn surface. The oxide layer forms rapidly in the contact area and grow in a layered structure, which will reduce the direct contact between the worn surface and the friction pair, then protect the worn surface [29]. When the oxide layer reaches a certain thickness, the adhesion between the oxide layer and the substrate will reduce and resulting in spalling. The bare matrix will contact directly with the friction pair again and cause the oxidation wear.

From Figure 7(a2–c2), it can be seen the worn surface roughness (Ra) of 15 wt.% VN/7075 and 20 wt.% TiB_2_-TiC_x_/7075 composites at 100 °C is 31.6 and 69.3% lower than that of Al 7075, respectively. The reason is that the hardness of VN and TiB_2_-TiC_x_ is not easy to decrease at 100 °C and can still support the matrix material well and resist the cutting effect on the grinding material. TiB_2_-TiC_x_ particles have a better supporting effect than VN particles due to its high content and hardness.

**Figure 8:**
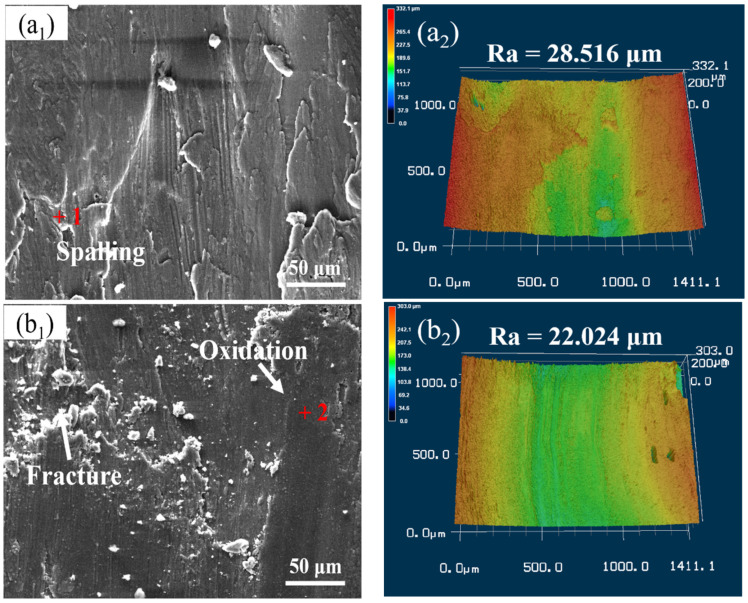

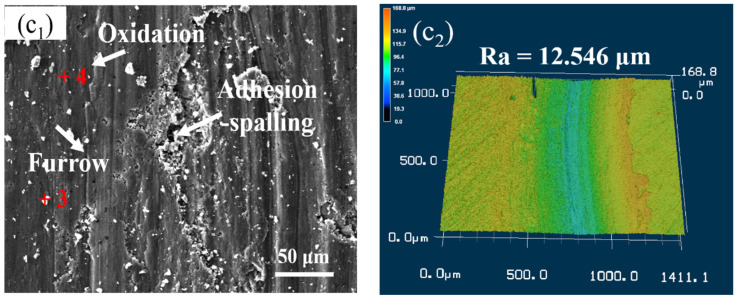
Worn surface analysis of samples after wear test at 200 °C. (**a1**) worn surface of Al 7075; (**a2**) 3D laser scanning topography of Al 7075; (**b1**) worn surface of 15 wt.% VN/7075; (**b2**) 3D laser scanning topography of 15 wt.% VN/7075; (**c1**) worn surface of 20 wt.% TiB2-TiCx/7075; (**c2**) 3D laser scanning topography of 20 wt.% TiB_2_-TiC_x_/7075.

The worn surface and 3D laser scanning morphology analysis of samples after wear test of Al 7075, 15 wt.% VN/7075, and 20 wt.% TiB_2_-TiC_x_/7075 composites at 200 °C are shown in Figure 8, and EDS results (wt.%) in regions 1–4 indicated in Figure 8 are listed in Table 5.

From Figure 8(a1–c1) and Table 5, there are micro furrows and spalling on the worn surface of Al 7075 (region 1), and there are aluminum oxides in the spalling. The worn surface of 15 wt.% VN/7075 composites has serious adhesive spalling (region 2), and there are aluminum oxide and titanium oxide in the worn surface. There are spalling debris, exfoliation pits and micro furrows on the worn surface of 20 wt.% TiB_2_-TiC_x_/7075 composites (region 3), and there are aluminum oxides and titanium oxides on the worn surface (region 4). Based on this, the wear mechanism of Al 7075 at 200 °C is spalling wear and oxidation wear. The wear mechanism of 15 wt.% VN/7075 and 20 wt.% TiB_2_-TiC_x_/7075 composites at 200 °C is oxidation wear, abrasive wear and adhesive wear. From Figure 8(a2–c2), it indicates that the worn surface roughness (Ra) of 15 wt.% VN/7075 and 20 wt.% TiB_2_-TiC_x_/7075 composites are 22.8 and 56% lower than Al 7075 at 200 °C. The oxidation process occurs easier at 200 °C due to the high temperature. The wear rate will decrease at the initial stage of oxide film formation, but the wear of composites will be aggravated when the oxide film breaks. Therefore, oxidation wear of the material at 200 °C is more serious than that at 100 °C, and there is oxide spalling on the worn surface [33]. In addition, the hardness of 15 wt.% VN/7075 composites decreases at higher temperature, which causes the supporting effect of the matrix to the oxide film on the worn surface to weaken and resulting in serious wear. Therefore, the reasonable combination of the stability and lubrication of the oxide film and the supporting function of the matrix can improve the wear properties of the material.

Compared with the wear surface roughness values of Al 7075, 15 wt.% VN/7075 and 20 wt.% TiB_2_-TiC_x_/7075 composites at room temperature, 100, and 200 °C, it can be seen that the wear surface roughness of the three materials increases with the increase of temperature. The friction coefficient of materials depending on many factors, while it is mainly related to the surface roughness. So the friction coefficient of the three materials increases due to the increasing wear surface roughness with temperature.

**Figure 9:**
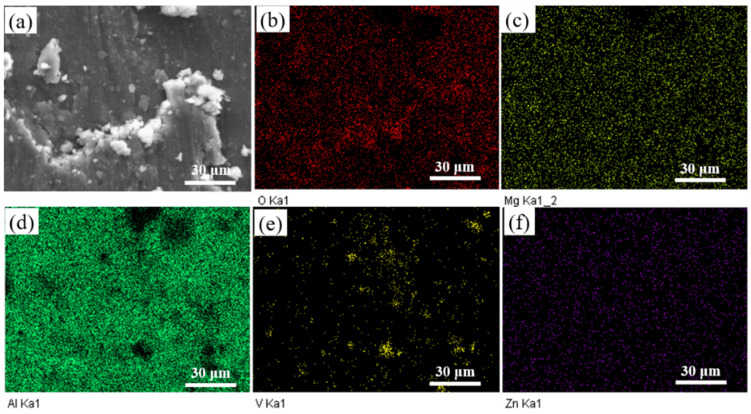
Elements distribution of 15 wt.% VN/7075 worn surface after wear test at 200 °C. (**a**) worn surface micromorphology; (**b**) O; (**c**) Mg; (**d**) Al; (**e**) V; (**f**) Zn.

Element distribution of 15 wt.% VN/7075 worn surface after wear test at 200 °C is shown in Figure 9. It can be seen the distribution of V and O in the worn surface is basically similar. It is further proved that there are vanadium oxides on the worn surface of 15 wt.% VN/7075 composites at 200 °C.

**Figure 10:**
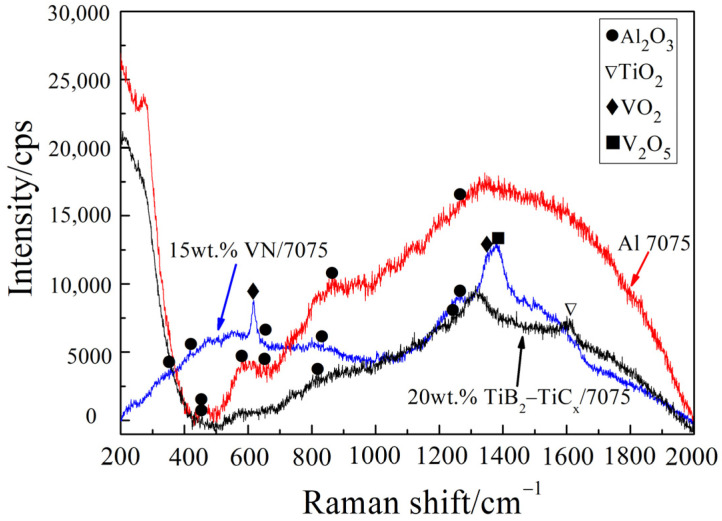
Raman measurement results of sample worn surface after wear test at 200 °C.

In order to further determine the type of compound on the worn surface at 200 °C, Raman spectroscopy was used to analyze the worn surface, as shown in Figure 10. The oxidation product of Al 7075 is Al_2_O_3_, the oxidation product of 15 wt.% VN/7075 composites is Al_2_O_3_, VO_2_, and V_2_O_5_, and the oxidation product of 20 wt.% TiB_2_-TiC_x_/7075 composites is Al_2_O_3_ and a small amount of TiO_2_.

At relatively high temperature (200 °C), oxidation occurs when the material moves relative to the contact surface. The oxide will participate in the relative motion process, which may control the friction and wear behavior of the moving parts. For example, some metal or nonmetal oxides (such as the oxides of Re, Ti, Mo, Zn, V, W, B, etc.) are easy to deform or shear in the friction process, and obtain lower friction coefficient (0.1–0.3) and wear rate [31]. By comparing the formation conditions of Ti and V oxides, it is found that Ti oxides are easier to form. At 200 °C, both composites are oxidized during the friction and wear process, and the formation of TiO_2_ is prior to VO_2_ and V_2_O_5_. These oxide layers are coated on the worn surface, which reduces the direct contact between the matrix and the abrasive material, thus improving the wear performance [33]. This is consistent with the variation of wear rate and the test results of the friction surface.

**Figure 11:**
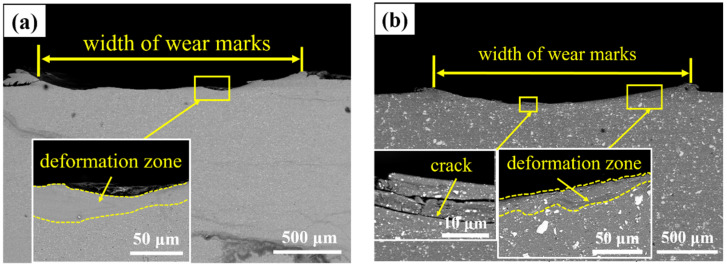

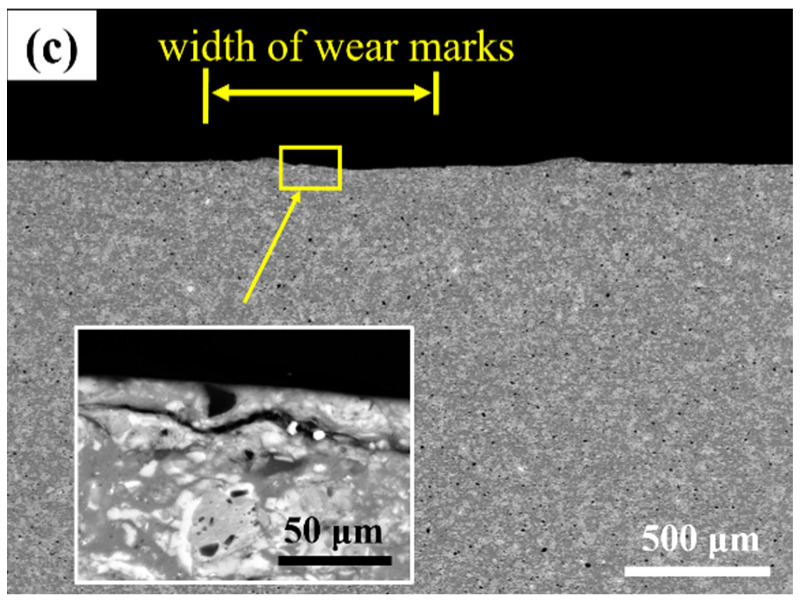
SEM micrograph of the clip plane of sample worn surface after wear test at 200 °C. (**a**) Al 7075; (**b**) 15 wt.% VN/7075; (**c**) 20 wt.% TiB_2_-TiC_x_/7075.

Figure 11 is the micrograph of the clip plane analysis of worn surface after wear test of Al 7075, 15 wt.% VN/7075, and 20 wt.% TiB_2_-TiC_x_/7075 composites at 200 °C, respectively.

As showed in Figure 11a, the worn width of Al 7075 alloy is the largest, and there is obvious deformation layer in the subsurface of the worn surface. This is due to the softening of the Al 7075 alloy at relatively high temperature. Figure 11b indicates that there are obvious cracks in the subsurface of the worn surface, which is because the worn surface temperature is higher and the compressive stress decays faster at 200 °C. The main action form of mechanical stress is shear stress, so microcracks parallel to shear stress are formed. Besides, a deformation layer in the subsurface can also be seen, while the range of deformation zone is smaller than those of Al 7075 alloy. The reason is that the hardness of the material is relatively low (although the hardness has been improved, it is not enough to resist the effect of wear compressive stress and shear force at 200 °C), the content of VN reinforcement phase is small (less than 20 wt.% TiB_2_-TiC_x_), so the plastic flow of Al 7075 matrix occurs at 200 °C. Moreover, there is a layer of oxide film on the worn surface, which reduces the friction coefficient of the material and protects the worn surface, thus improving the wear performance. Figure 11c is the morphology of 20 wt.% TiB_2_-TiC_x_/7075 composites. There are only shallow cracks near the worn surface and almost no deformation zone. The worn surface is smooth, and the oxide film can play a protective role. At the same time, TiB_2_-TiC_x_ phase becomes the main bearer of the load, which improves the hardness of the material and supports the oxide film better. Therefore, the wear resistance of the material has been improved.

In the atmospheric environment, the oxide film on the worn surface of composites can play a certain role of friction reduction, and also can protect the worn surface and reduce the amount of wear even at medium and low temperatures. The high hardness enhancement phase can improve the hardness of the matrix and better support the oxide layer to resist the cutting of the grinding material. The complexity of the wear process lies in the combined action of various mechanisms. Therefore, the stability and lubrication of the oxide film properly combining the strong supporting role of the matrix can improve the wear properties of the material.

## 4. Conclusions

In this paper, Al 7075 alloy, 15 wt.% VN/7075 composites, and 20 wt.% TiB_2_-TiC_x_/7075 composites were prepared by ball milling with subsequent hot-pressing sintering. The microstructure, hardness, and wear properties at room temperature—200 °C of Al 7075-based composites were discussed. The results are as following:

1. Al 7075 alloy sintered after hot-pressing at 520 °C are mainly composed of Al (Zn, Mg, Cu) matrix phase and rich copper phase. The phase composition of 15 wt.% VN/7075 composites is mainly Al (Zn, Mg, Cu) solid solution and VN phase. The grain uniformity degree is 0.25, indicating that the distribution of VN particles is uniform. For 20 wt.% TiB_2_-TiC_x_/7075 composites, Ti_3_B_4_ and B_4_C appear besides Al, TiB_2_, and TiC_x_ phases, and the microstructure with the grain uniformity degree of 0.13 is relatively uniform.
2. The hardness of the three materials is 20 wt.% TiB_2_-TiC_x_/7075 composites (270.2 HV) > 15 wt.% VN/7075 composites (119.5 HV) > Al 7075 (81.8 HV). Compared with Al 7075 alloy, the hardness of 20 wt.% TiB_2_-TiC_x_/7075 composites and 15 wt.% VN/7075 composites increased by 230.3 and 46.1%, respectively.
3. At medium and low temperatures, the friction coefficient and volumetric wear rate of the three materials are increased with the increase of temperature. At the same temperature, the friction coefficient of 15 wt.% VN/7075 composites is the smallest. Compared with Al 7075, it reduced by 25.1 (room temperature), 12.7 (100 °C), and 9.8% (200 °C), respectively. The volumetric wear rate of 20 wt.% TiB_2_-TiC_x_/7075 composites is the lowest. Compared with Al 7075, it reduced by 46.2 (room temperature), 52.6 (100 °C), and 34% (200 °C), respectively.
4. With the increase of temperature, the wear mechanism of Al 7075 changed from spalling wear at room temperature to oxidation wear and adhesive wear at medium and low temperature. The wear mechanism of 15 wt.% VN/7075 and 20 wt.% TiB_2_-TiC_x_/7075 composites changed from abrasive wear at room temperature to oxidation wear, abrasive wear, and adhesive wear at medium and low temperature.

## Figures and Tables

**Table 1:** Ball milling parameters of 3Ti + B_4_C→2TiB_2_ + TiC.

Name	Parameters
Milling balls and bowl	304 stainless steel
Diameter of milling ball	5 mm
Speed	300 r/min
Mass ratio of ball to material	10:1
Milling time	40 h
Process control agent	Stearic acid (10% of total mass)

**Table 2:** EDS results (wt.%) in regions 1–5 indicated on Figure 1a–c.

Region	Al	Mg	Zn	Ti	B	C	O	Fe	Cu	V	N
1	69.98	1.27	1.41	-	-	-	17.94	-	9.43	-	-
2	1.08	-	-	-	-	-	-	-	-	67.45	31.47
3	86.84	1.32	1.25	-	-	-	7.97	-	1.04	1.58	-
4	72.61	1.70	4.22	8.15	5.30	1.86	4.74	-	1.42	-	-
5	58.55	2.36	2.59	18.03	-	8.70	4.34	2.84	0.97	-	-

**Table 3:** EDS results (wt.%) in regions 1–3 indicated in Figure 5.

Region	Al	O	Mg	Zn	Cu	V	N	Fe	Si	Ti	B	C
1	94.95	-	1.26	2.72	1.08	-	-	-	-	-	-	-
2	47.79	24.26	1.76	2.11	1.03	20.23	1.26	0.62	-	-	-	3.43
3	25.49	5.72	0.84	1.95	1.69	-	-	0.50	0.42	3.57	57.81	-

**Table 4:** EDS results (wt.%) in regions 1–5 indicated in Figure 7.

Region	Al	O	Mg	Zn	Cu	V	N	Fe	Si	Ti	B	C
1	74.37	16.56	2.44	5.19	1.45	-	-	-	-	-	-	-
2	61.39	13.28	1.34	6.77	1.83	14.39	-	-	-	-	-	-
3	44.75	47.46	1.40	2.49	-	3.89	-	-	-	-	-	-
4	53.13	34.35	1.58	3.69	0.99	5.82	0.44	-	-	-	-	-
5	53.82	23.95	2.09	3.32	1.10	-	-	0.28	0.79	8.87	8.87	-

**Table 5:** EDS results (wt.%) in regions 1–4 indicated in Figure 8.

Point	Al	O	Mg	Zn	Cu	V	N	Fe	Si	Ti	B	C
1	58.73	31.91	1.89	4.22	1.30	-	-	-	-	-	-	-
2	15.41	28.07	0.67	1.00	-	48.93	5.95	-	-	-	-	-
3	45.51	44.85	1.58	2.31	-	-	-	0.85	-	3.77	-	1.50
4	44.95	23.23	3.39	2.85	-	-	-	1.06	-	13.41	-	11.11

## Data Availability

All the data is available within the manuscript.
